# Gene editing and CRISPR-dependent homology-mediated end joining

**DOI:** 10.1038/s12276-025-01442-z

**Published:** 2025-07-31

**Authors:** Brian L. Ruis, Anja K. Bielinsky, Eric A. Hendrickson

**Affiliations:** 1 Department of Medicine, University of Virginia, Charlottesville, VA USA; 2 Department of Biochemistry and Molecular Genetics, University of Virginia, Charlottesville, VA USA

**Keywords:** Gene targeting, Biotechnology

## Abstract

Gene editing is the intentional modification of a genetic locus in a living cell and is used for two general applications of great importance and wide interest. One is the inactivation of genes (‘knockouts’), a process utilized to delineate the loss-of-function phenotype(s) of a particular gene. The second application (‘knock-ins’) is essentially the process of gene therapy, which predominately involves correcting a pre-existing mutated allele(s) of a gene back to wild-type to ameliorate some pathological phenotype associated with the mutation. Importantly, although these applications are conceptually exact reciprocal opposites of one another, they are achieved via mechanistically different pathways. In the case of knockouts, breakage (usually in the form of double-stranded breaks) of the chromosomal DNA at the site of targeting is used to engage a repair process (nonhomologous end joining) that is error prone. The ensuing repair frequently results in insertions/deletions at the cleavage site, which, in turn, results in out-of-frame mutations and, hence, a knockout of the gene in question. In the case of knock-ins, breakage (again, usually in the form of double-stranded breaks) of the DNA is used to engage a repair process (homology-dependent repair/recombination) in which homologous sequences between an incoming donor DNA (containing new genetic information) and the chromosomal DNA are exchanged. Although homology-directed repair was known to predominate in bacteria and lower eukaryotes, the competing process of nonhomologous end joining predominates in higher eukaryotes and was presumed to prevent the use of knock-in gene editing in human somatic cells in culture. A series of molecular and technical advances disproved this notion but still resulted in a process that was cumbersome, labor intensive, highly inefficient and slow. In 2013, however, a new RNA-programmable nuclease, CRISPR–Cas9 was described that has revolutionized the field and made gene editing accessible to anyone with even a rudimentary knowledge of molecular biology. Thus, gene editing in a wide variety of model organisms, as well as human somatic cells in culture, has become not only extremely feasible but also extremely facile, and it harbingers a golden age for directed mutagenesis, directed evolution and improvements in gene therapy.

## Gene editing revolutionizes therapy and research techniques

In recent years, clustered regularly-interspersed short palindromic repeats and associated protein 9 (CRISPR–Cas9) has revolutionized gene editing, allowing scientists to make precise changes to DNA. Researchers developed CRISPR–Cas9, which acts as molecular scissors guided by RNA to target specific DNA sequences. This method is faster and more accurate than previous techniques. CRISPR–Cas9 can create ‘knockouts’ by disrupting genes or ‘knock-ins’ by adding new genetic material. It relies on two main DNA repair processes: nonhomologous end joining, which can be error prone, and homology-directed repair, which is more precise but less common. Scientists are working to improve homology-directed repair efficiency for better gene editing outcomes. The technology has vast potential for treating genetic diseases, but challenges remain, such as ensuring precision and avoiding unintended effects. Future research aims to refine CRISPR–Cas9 for safer and more effective therapies.

This summary was initially drafted using artificial intelligence, then revised and fact-checked by the author.

## Introduction

All researchers who have for a long time been engaged in science are able to recall technological discoveries that substantially improved their day-to-day laboratory existence. Examples such as Southern blotting for detecting specific fragments of DNA^1^ or the yeast two-hybrid assay^2^ for the identification of protein–protein interactions would fall into this category. Much less frequently, there are technological discoveries that result in advancements not just for a particular field but literally change the way that science is being performed in many fields. The description in 1975 by Georges Kohler and Cesar Milstein of the production of hybridomas and their ability to produce monoclonal antibodies certainly ranks in this category^3^. The ability to identify any protein in any model organism was profoundly useful to researchers world wide. As influential and, perhaps, even more transformative was the discovery and development in the mid- to late 1980s by Kary Mullis of the polymerase chain reaction (PCR)^4^. At that time, most molecular biologists spent vast amounts of their day-to-day professional lives isolating and grafting small fragments of DNA—usually grown up in bacteria or isolated out of polyacrylamide gels—to one another to create some useful recombinant DNA molecule. PCR seismically altered the way that almost all researchers carried out science, such that it was now possible to do the same science but at a rate that was exponentially faster and with a precision that was orders of magnitude better.

Despite these reoccurring breakthroughs that permeate and drive scientific advancements, it is still difficult to comprehend and appreciate the enormity of the metaphorical tsunami of excitement and change that has resulted from the application in 2013 of clustered regularly-interspersed short palindromic repeats and associated protein 9 (CRISPR–Cas9) to the field of mammalian gene editing^5–7^. In the past decade there has been a nearly unfathomable outpouring of publications, review articles (this review will certainly be just one of hundreds published this year on CRISPR–Cas9), major workshops and scientific conferences, discussions in the general press, even an international summit organized by a bevy of Nobel laureates (to lobby in on the ethics of human gene editing^8,9^), its designation by the journal *Science* as ‘Breakthrough of the Year’^10^ and, finally, the acknowledgement of its importance by the awarding of Nobel Prizes in 2020 to Emmanuelle Charpentier and Jennifer Doudna. If scientific discoveries were nuclear disasters, CRISPR would be far bigger than Chernobyl.

As will be discussed below, the Cas9 component of the CRISPR system is essentially an RNA-programmable restriction enzyme. At first reflection, this does not seem to be that earth-shattering of an activity nor a particularly useful one. The limitation of most restriction enzymes, however, is that their recognition sequence is relatively small: usually 4 or 6 bp. As a consequence, all classic restriction enzymes recognize so many sites in the human genome that their utility is extremely constrained. Cas9, however, is dependent upon a ~100-nt-long (which includes a ~20-nt-long targeting stretch) single guide RNA (sgRNA) molecule. Because of the stability of complementary RNA–DNA hybridization and because of the length of the RNA molecule, each Cas9 is essentially directed to one and only one location in the human genome (for discussions of CRISPR’s off-target activity, the reader is referred to any number of reviews devoted predominately to this topic^11,12^). It is this ability—the ability to do meaningful chemistry at a precisely defined locus in the human genome—that provides CRISPR–Cas9 with its unique usefulness. For the purposes of this review, we will provide a short history of the field as well as give a brief overview of the pathways relevant for gene editing. In the main part of the review, we will attempt to summarize a new area where CRISPR–Cas9 has been applied and the current attempts to improve and expand its utility for gene therapy.

## Relevant background

### A brief history of gene editing

Bacteria and, especially, yeast owe their popularity as model systems to the ease with which researchers could carry out forward genetic screens and to the ease with which researchers could use reverse genetics to precisely alter these organisms’ genomes. In higher organisms, forward genetic screens were logistically (or ethically) often impractical and, thus, reverse genetic approaches were the only functional way to carry out the search for new genes and/or genome modifications. The first published report of gene targeting in human cells occurred four decades ago when Smithies et al. attempted to disrupt the β-globin locus in the human EJ bladder carcinoma cell line^13^. No correctly targeted cell lines were obtained, but the same authors subsequently successfully targeted the human β-globin locus in a human:mouse hybrid cell line. Several years later, Jasin et al. reported the first isogenic targeting of an endogenous locus (the CD4 gene) in a completely human (the T-lymphocyte JM) cell line^14^. Although valuable as proof-of-principle experiments, the reported frequency of 10^−7^ to 10^−8^ made this approach beyond daunting from a practical point of view except for the most masochistic (or stubborn) of researchers. The development of viral vectors (such as recombinant adeno-associated virus (rAAV)) to facilitate gene targeting greatly improved on these frequencies but were not widely adopted because they required some knowledge of virology and were still cumbersome in their design (reviewed by refs. ^15,16^). Taking a cue from studies done earlier in yeast, the laboratory of Maria Jasin subsequently made the important demonstration that the introduction of a double-stranded break (DSB) at the chromosomal locus of interest increased the frequency of gene targeting to a now useful frequency of 10^−3^ to 10^−4^ (refs. ^17,18^). Again, while valuable as a proof of principle, the technical problem with these experiments was that they were carried out with a fungal meganuclease that had no naturally occurring sites in the human genome. To be of utility for gene editing, a researcher needs to be able to direct a DSB precisely (often to the nucleotide) to their chromosomal locus of interest. Thus, the field scrambled to devise programmable nucleases, which ultimately resulted in the development and use of zinc finger nucleases (ZFNs) in the early 2000s^19^. While some ZFNs proved quite useful and even made their way into the clinic, many were difficult to produce and then showed only marginal and/or promiscuous cleavage activity. The field received what looked like a remarkable breakthrough, with the identification and use of transcription activator-like effector nucleases (TALENs)^20^. TALENs were easier to produce than ZFNs and appeared to have improved cleavage specificity. Unfortunately, TALEN production was still rather laborious and required that a unique TALEN needed to be manufactured for every site that a researcher wanted to engineer. Thus, when it was demonstrated in 2013 that CRISPR–Cas9, which had been well-documented to be an RNA-programmable nuclease that bacteria used as part of a primitive adaptive immune system^21^, could be reprogrammed to work in mammalian cells the last major barrier to efficient human cell gene editing was hurdled. In the intervening decade, CRISPR–Cas9 has been utilized by thousands of laboratories to carry out a myriad of interesting biology, and it is clearly the preferred molecular tool to carry out just about any reverse genetic experiment that can be envisioned.

### NHEJ

**Fig. 1:**
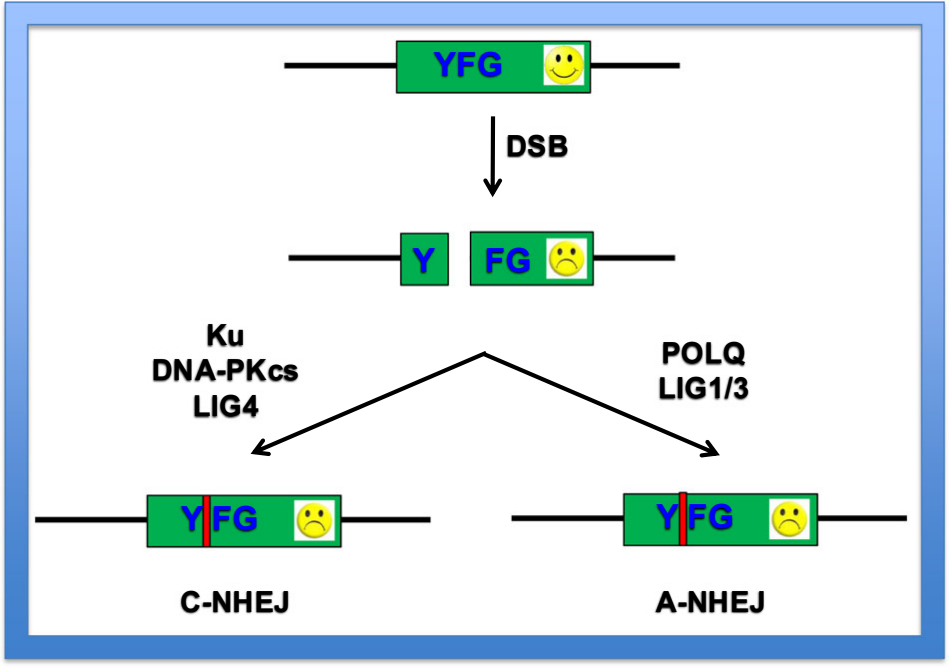
Schematic of the NHEJ pathways. A DSB is introduced into a functional (smiley face) locus of human genomic DNA designated as YFG, which temporarily inactivates it (frowny face). The chromosomal DSB can be repaired by either of two end joining pathways: either C-NHEJ, which requires the factors Ku, DNA-PKcs and LIG4, or via A-NHEJ, which requires the factors POLQ and LIG1/3. Because both of these pathways can be error prone, they can generate insertions and deletions (‘indels’, red bar) that cause out-of-frame mutations and result in the permanent inactivation of the gene (frowny face) following completion of repair.

A double-stranded piece of linear DNA introduced into a mammalian cell can be incorporated into that cell’s genome by either nonhomologous end joining (NHEJ) or homology-directed repair (HDR) (reviewed by refs. ^22,23^). Bacteria and lower eukaryotes utilize HDR almost exclusively for the uptake of foreign DNA. In higher eukaryotes, however, integration proceeds more frequently by a process that does not require extended regions of homology. Specifically, mammalian cells—and humans in particular—have evolved a highly efficient ability to join nonhomologous DNA molecules together^24^. In their seminal work on gene targeting, Capecchi and coworkers showed that although somatic mammalian cells can integrate a linear duplex DNA into corresponding homologous chromosomal sequences using HDR, the frequency with which recombination into nonhomologous sequences occurred was at least 1000-fold greater^25^. This NHEJ pathway, which is generally referred to as the classical (or C-NHEJ) pathway, appears to be predominately active during the G_1_/early S phase of the cell cycle^26,27^. When a DSB is introduced into a gene (your favorite gene, hereafter YFG) the broken ends are bound by a heterodimeric (Ku70:Ku86) protein complex termed Ku, that, in turn, acts to recruit a kinase, the poorly-named DNA-dependent protein kinase complex subunit (DNA-PK_cs_)^28^ (Fig. 1). Activation of the kinase results in the formation of a synaptic complex^29^, which recruits downstream factors that are involved in synapsis and end trimming/polishing^22^. The ends are ultimately sealed back together by DNA ligase 4 (LIG4). Because of the end trimming/polishing step, nucleotides can be inserted or deleted (aka ‘indels’) at the site of repair and if this occurs within the coding region of a gene it can result in out-of-frame, loss-of-function mutations (Fig. 1). Finally, it has been appreciated that there is at least one additional subpathway of NHEJ (often referred to as ‘alternative’ NHEJ or interchangeably as A-NHEJ or A-EJ^22,30^) that exists in mammalian cells. Operationally, A-NHEJ is somewhat nonhelpfully defined as the end joining that occurs in the absence of C-NHEJ factors/activity. Despite a decade’s worth of intense research, however, it is still not clear whether A-NHEJ is a single pathway. Thus, because many (albeit not all) of the A-NHEJ-mediated repair events often contain microhomology at their junction, they are sometimes also referred to as microhomology-mediated end joining (MMEJ) products^31,32^. The equivalence of A-NHEJ with MMEJ is, alas, vague. Jeremy Stark’s laboratory has suggested that events containing three or more nucleotides of microhomology can formally be deemed as MMEJ, whereas events with only one or two nucleotides of microhomology are better defined as generic C-NHEJ/A-NHEJ events^33^. To complicate matters even more, at least a portion of A-NHEJ/MMEJ events are generated during mitosis due to their reliance on DNA polymerase theta (POLQ), an enzyme whose activity is significantly upregulated during that stage of the cell cycle^34^. These events are thus often referred to as theta-mediated end joining, but an understanding of the distinctions (if indeed there are any) between A-NHEJ/A-EJ, MMEJ and theta-mediated end joining are currently not clear^35^, and thus, for the purposes of this review, all of these will collectively be referred to as A-NHEJ. A-NHEJ repaired ends are rejoined by either DNA ligase 1 or DNA ligase 3 (LIG1/3) (Fig. 1). Neither of these pathways are precise and consequently the products of repair often contain indels that ablate any corresponding open reading frame. Given that NHEJ-mediated DSB repair (especially A-NHEJ) can be error prone, an attribute that bacteria and lower eukaryotes can ill afford, the increased percentage of noncoding DNA in higher eukaryotes may have facilitated the evolution of these imprecise pathways. In summary, mammals are different from bacteria and lower eukaryotes in that DSB repair proceeds primarily through an NHEJ repair pathway. Moreover, and most importantly for this review, it is important to appreciate that NHEJ must be overcome to facilitate gene targeting, and this can only occur when the incoming DNA is shunted into the HDR pathway.

### HDR

**Fig. 2:**
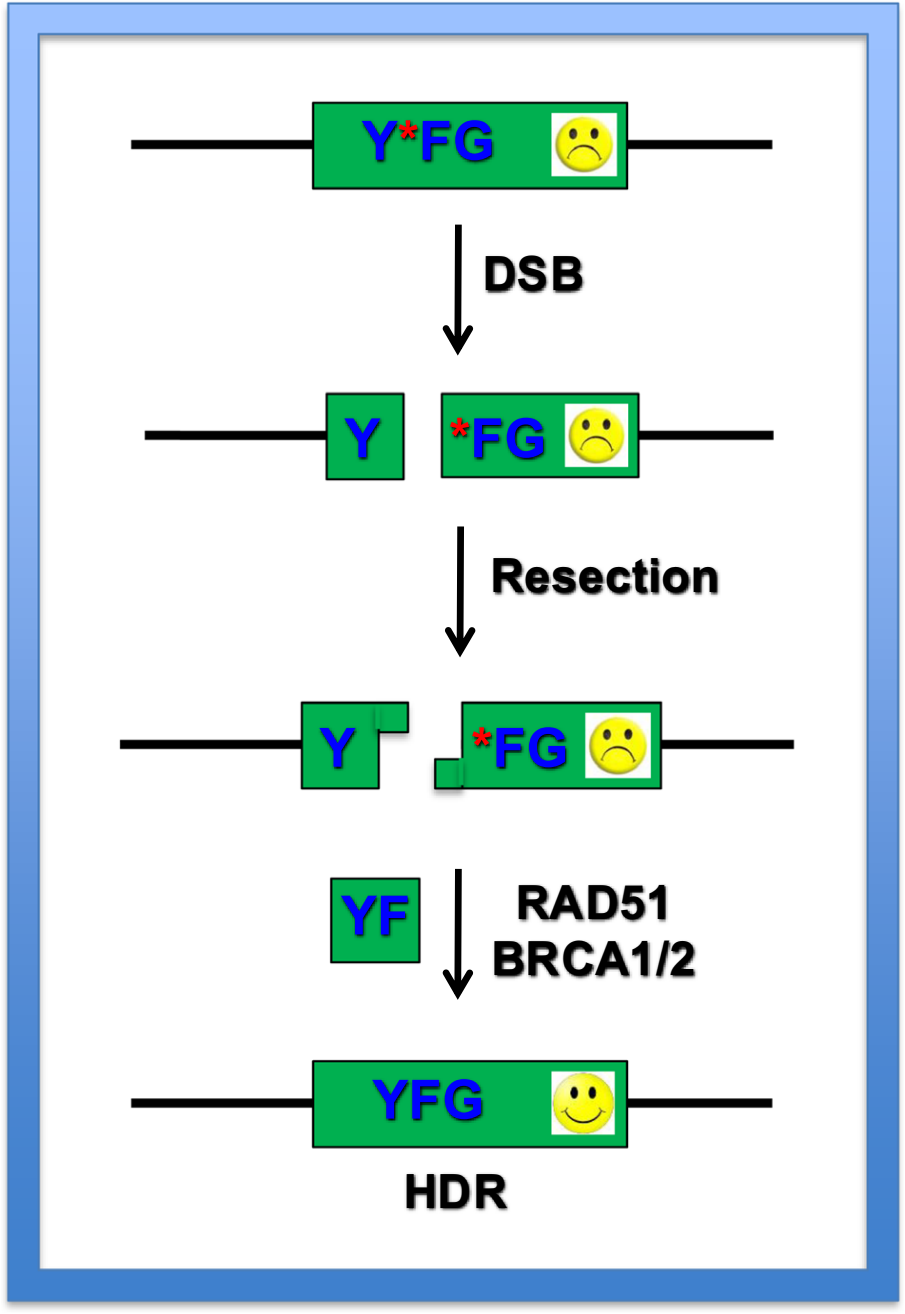
Schematic of the HDR pathway. An example that illustrates the principle of gene therapy is shown. A DSB is introduced into a nonfunctional (frowny face) locus of human genomic DNA designated as YFG. YFG is nonfunctional because it contains some sort of mutation (red asterisk). The chromosomal DSB undergoes resection to yield single-stranded 3′-OH overhangs. These resected ends are platforms for the recruitment of a bevy of recombinational repair proteins, principally including RAD51 and BRCA1/2. If a donor DNA (green YF rectangle) is present, these recombinational proteins facilitate the replacement of the chromosomal YF sequences with the donor sequences. This results in the restoration of a functional chromosomal YFG (smiley face) following completion of repair.

In HDR (reviewed by ref. ^36^), the DNA ends of the DSB are resected to yield 3′-single-stranded DNA overhangs (Fig. 2). The resulting overhangs are then coated by radiation sensitive 51 (RAD51; Fig. 2). RAD51 is the key strand exchange protein in HDR. It is essential for the homology searches on the target DNA—that is, often the entire human genome—that are required to localize the incoming DNA to its specific, cognate chromosomal counterpart. In humans, there are at least seven Rad51 family members, and almost all of them have been implicated in some aspect of HDR and also in human disease^37^. Strand invasion into the homologous chromosomal sequence or donor DNA probably requires a bevy of accessory proteins that includes Breast Cancer Alleles 1 and 2 (BRCA1/2). The resolution of this repair intermediate generates a modified chromosome in which the original chromosomal sequences have been replaced by sequences present on the incoming donor DNA. If the donor DNA contains wild-type sequences, then any pre-existing mutations in the chromosomal DNA will be corrected, and the function of the gene will be restored. Historically, large (generally many kilobase pairs long) double-stranded DNA (dsDNAs) were used for donors due to the requirements for long regions of homology to facilitate a canonical HDR reaction^38^. However, dsDNA is a very potent activator of the human innate immune system (which apparently sees the donor dsDNA as the product of a viral infection), and this fact has limited the use of dsDNA donors. Consequently, many therapeutic protocols instead try to utilize small (general just 100 nucleotides long) oligodeoxynucleotides (ODNs). Fortunately, some of the HDR subpathways can very efficiently utilize ODNs to carry out repair/recombination. While ODNs generally circumvent the deleterious effects associated with immune activation caused by dsDNA, they nonetheless have the downside that they cannot encode significant amounts of genetic information, which is usually restricted to about 30 nucleotides per repair/recombination event^39^. As is the case with NHEJ, the use of these HDR subpathways is probably highly cell cycle^40^ and tissue variable^36^, and one of these subpathways (homology-mediated end joining; HMEJ) will be discussed in depth below. In summary, human somatic cells express all the gene products needed to carry out gene targeting. These events occur, however, at very low frequency due to the preferred usage of NHEJ and alternative pathways of HDR.

## The many uses of CRISPR–Cas9

### Knockouts

In the three decades of gene editing preceding the development of CRISPR–Cas9 as a gene editing tool approximately 150 genes in the human genome had been modified by simple DNA transfections, rAAV infections or through ZFN- or TALEN-mediated gene targeting^41^. Overnight, the development of CRISPR–Cas9 enlarged this number to essentially every gene in the human genome (~20,000). Needless to say, characterization of all of these knockouts has lagged behind their generation, but it is only a matter of time before a phenotypic characterization of these modifications occurs. Indeed, the National Institutes of Health (NIH) established the Somatic Cell Genome Editing (SCGE) consortium in 2018, which was specifically tasked with enhancing every aspect of gene editing to enable the development of a data warehouse for the effect of editing every gene expressed in human somatic cells. The second phase of that consortium started last year, and its goal is to now translate that phase one data into clinical therapies.

The simplest but often most important modification that a researcher can make is a knockout of a gene of interest. This type of mutation allows a researcher to assess the loss-of-function phenotype of a gene. CRISPR–Cas9 accomplishes this exactly the way that ZFN- and TALEN-mediated modification did, by relying on the abundant cellular NHEJ systems (Fig. 1) to inaccurately repair the resulting DSB. Thus, the empirical observation is that indels occur at high frequency at the sites of CRISPR–Cas9 cleavage^5–7^. If the locus of editing is an exon, these alterations invariably cause frameshift mutations that inactive the gene of interest. The great improvement that CRISPR–Cas9 provides over the earlier technologies is twofold. First, it is so efficient that often biallelic modification occurs. Thus, before CRISPR–Cas9, rAAV-, ZFN- or TALEN-mediated strategies invariably resulted in only one of two homologous chromosomes in a diploid mammalian cell becoming modified. This required the isolation and characterization of the intermediate heterozygous cell line, before an entirely new round of transfection/infection could be performed to obtain null cell lines. By contrast, CRISPR–Cas9 is so efficient that modification of both chromosomes occurs simultaneously at high frequency^42^.

The fact that CRISPR–Cas9 can make biallelic mutations at high frequency has an additional advantage: it has allowed for the construction of whole-genome knockout libraries. Thus, over a decade ago, researchers constructed knockout libraries for every nonessential gene in the yeast genome. These knockout libraries allowed yeast researchers to ask global questions about cellular regulation that were simply not possible when single mutants were analyzed one at a time^43–45^. The construction of the yeast knockout libraries was, however, vastly simplified by the haploid nature of yeast, where disruption of only a single allele was needed to obtain a null phenotype. Similar libraries for potentially more relevant model systems (for example, zebrafish, mice and human somatic cells) were deemed impractical because the diploid nature of these organisms would require biallelic disruption to reveal phenotypically recessive traits. In 2014, taking advantage of the efficacy of CRISPR–Cas9, CRISPR-based whole-genome knockout libraries were described by two groups^46,47^. These groups constructed lentiviral vectors that permitted the expression of ~70,000 different sgRNAs capable of inactivating 18,000 human genes. The libraries and technology were then successfully used to identify novel genes involved in rapidly accelerated fibrosarcomas (RAF) inhibition^47^, mismatch DNA repair and topoisomerase poison sensitivities^46^ and tumor necrosis factor (TNF) signaling^48^. In conceptually parallel work, the ~2000 essential genes in the human genome were identified by using CRISPR–Cas9 to inactivate every human gene and then characterize the genes that were depleted in the screen^49,50^. These types of unbiased, whole-genome screens are completely poised to revolutionize human somatic cell genetics and have subsequently been used in hundreds, if not thousands, of laboratories since their development.

The second big improvement is that CRISPR–Cas9 can be multiplexed. Since Cas9 is regulated at the level of guide RNAs, and each sgRNA is only ~100-nt-long, multiple sgRNAs to multiple targets can be simultaneously expressed in the same cell facilitating simultaneous, biallelic conversions. Thus, the construction of doubly, triply or multiply modified genetically modified cells/organisms, which previously would have taken years to painstakingly construct, can now be done in a single, quick experiment. Some of the reported successes have been rather spectacular. In one instance, five different genes in mouse embryonic stem cells were simultaneously modified^51^, most of them biallelically. One of the more impressive feats was that all 62 endogenous retroviruses in a porcine cell were coordinately inactivated^52^ to derive pigs more suitable for human xenotransplantation.

### Knock-ins

As elaborated above, the efficacy of generating knockout mutations using CRISPR–Cas9 is exceptionally high. This outcome is the direct result of two factors: the high efficiency of cleaving genomic DNA by CRISPR–Cas9 and the predominant use of error-prone NHEJ in higher eukaryotes. By contrast, the ability to alter a genome exactly to the researcher’s choosing is generally referred to as a knock-in. This type of modification is extremely attractive to gene therapists and to anyone trying to precisely alter a genome with the goal of achieving specific phenotypic outcomes. Knock-in mutations, however, rely not on NHEJ but on HDR. Consequently, achieving knock-in mutations in higher eukaryotes, while possible, is nonetheless a low frequency event. Importantly, the frequency of generating knock-in mutations using rAAV^53^, ZFNs^54^, TALENs^55^ and even CRISPR–Cas9^51,56^ is generally in the range of only a few percent and suggests, therefore, that the rate-limiting step is not nuclease-dependent genomic cleavage but the low frequency of endogenous HDR activity. As a consequence, there have been a bevy of reports in which investigators have used either small molecule inhibitors of NHEJ^57,58^ or activators of HDR^59,60^ to enhance CRISPR–Cas9-mediated knock-ins (reviewed in ref. ^61^). Because of the anticipated utility of gene editing, a concerted effort is ongoing in many laboratories to improve these preliminary modifications and to develop novel approaches focused either on delivery of the donor DNA^62^ or modulation of the cellular DSB repair systems^61^. In our laboratory, one of the most useful tools to augment knock-in gene editing is a mysterious proprietary reagent termed ALT-R HDR Enhancer V2 (purported to contain a C-NHEJ inhibitor^62^), available from Integrated DNA Technologies. A consistent, robustly large increase in correct HDR-mediated knock-in outcomes has been achieved when the ALT-R HDR Enhancer V2 has been utilized with single-stranded ODN donors (Table 1).

**Table 1:** Impact of the IDT’s proprietary HDR enhancer V2 on ssDNA oligonucleotide donor incorporation.

Cell line	Gene	Donor	Homology arm length	−HDR EnhV2	+HDR EnhV2
HCT116	ZEB1	tCTS ssODN*	55 nt each	33%	79% (2.4X)
HeLa	HIBCH	tCTS ssODN*	50 nt each	12%	71% (5.9X)
HCT116	PIGA	ssODN	48 nt each	1%	44% (44X)
HCT116	XPA	tCTS ssODN*	50 nt each	17%	76% (4.5X)
HeLa	PGLS	tCTS SSODN*	50 nt each	6%	37% (6.2X)
HCT116	CD81	tCTS ssODN*	50 nt each	14%	69% (4.9X)

tCTS ssODN is an ssODN modified to contain a truncated 5′ Cas9 target sequence as defined by Shy et al.^119^.

An additional important application of knock-in technology is not in altering the primary sequence of a gene but in appending onto the gene (generally at the N- or C-terminus) a useful epitope (also known as epitope tagging). Epitope tagging (using a plethora of either fluorescent tags or epitopes readily recognized by commercially-available antibodies) has a myriad of uses including but certainly not limited to (1) determining the cellular location or the cell cycle regulated expression of a gene, (2) permitting immunological detection of a protein that is inherently nonimmunogenic, (3) generating a protein that can be used in chromatin immunoprecipitation or similar assays and (4) generating proteins whose expression can be tightly and reversibly regulated^63–66^. Again, the utility of CRISPR–Cas9 to make precise DSBs in the human genome where cassettes containing these useful epitopes can be inserted in-frame has greatly enhanced this technology^67,68^.

### Even a ‘dead’ Cas9 can be exceptionally useful in CRISPRa and CRISPRi

In its native conformation, Cas9 is a double-stranded nuclease that makes a blunt DSB at a target locus. It does so through the concerted action of two catalytic cores that coordinately make juxtaposed single-stranded breaks on opposing strands^69^. Molecular biologists have taken advantage of this fact to produce Cas9 variants in which either one of the active sites has been mutated (referred to as ‘Cas9 nickases’ or ‘nCas9’) or both active sites have been mutated (referred to as ‘dead Cas9’ or ‘dCas9’). dCas9 is devoid of DNA cleavage activity, an activity that this review has been extolling from the introductory sentence. Despite this—or, actually, more accurately, because of this—dCas9 has proved exceptionally useful as a genome-engineering tool as well. This is because that while dCas9 cannot cleave DNA, it still can localize (via the information encoded in the sgRNA) with pinpoint accuracy to any location within a genome. Researchers have taken advantage of this feature to engineer and then express chimeric versions of Cas9 that are fused to either transcriptional activation domains (CRISPR activation or CRISPRa) or transcriptional repression domains (CRISPR inhibition or CRISPRi) to selectively turn individual genes on or off, respectively^70,71^ (reviewed in refs. ^72,73^). The elegance of these systems is that it allows for complicated manipulation of a transcriptional process without physically altering the genomic DNA sequence. An additional attraction is that the systems are often fully reversible by using either light or small molecules to regulate the transcriptional activation/inhibition^74,75^. Much of this technology is still being improved/optimized, but essentially, any epigenetic modification (for example, histone methylation, histone acetylation or protein ubiquitination) for which the enzymatic activity has been biologically defined can be recapitulated with the CRISPRi/CRISPRa systems.

## Is CRISPR–Cas9 safe to use?

In its native conformation, CRISPR–Cas9 efficiently makes a DSB at a target locus. However, it is perhaps one of the few inviolate rules of biology that no biological process works perfectly all the time. Thus, almost simultaneously with the joyous hullabaloo that accompanied the initial successes with CRISPR–Cas9 came equally vocal, seasoned rejoinders of caution. In particular, it is well appreciated by radiation biologists everywhere that even a single mis-repaired or unrepaired DSB is either mutagenic or lethal, respectively. Thus, if CRISPR–Cas9 makes DSBs at even a low frequency at off-target sites, the risk for unwanted genome alterations and/or toxicity is great. For basic research applications, such off-target effects could certainly confound data interpretations and/or lead to spurious phenotypes, whereas in clinical research applications, such off-target effects could be outright lethal. Indeed, some early publications suggested that ancillary DSBs could not only occur but that in rare instances they could occur so often as to negate the utility of the system^76–78^. Most of these critical reports, however, proved not to be generalizable. Indeed, the empirical observation that live mice could be generated at very high frequency from CRISPR–Cas9-modified mouse embryonic stem cells demonstrated that if off-target modifications were occurring, they were happening at such a low level as to not impede the very sensitive process of murine development^51,56^. Subsequently, better computational programs for designing sgRNAs^79^, a better understanding of the specificity attributes of the targeting sequence within the sgRNA^80^ and how the sgRNA interacts with the target sequence^81^, greatly improved the efficacy for cleavage at the desired locus, although extremely sensitive whole-genome sequencing applications could still detect rare off-target activity^82^. Subsequently, however, two groups independently reported the development of molecularly evolved variants of Cas9 with enhanced binding and cleavage specificities such that these second-generation Cas9s had virtually no detectable (even by sensitive whole-genome sequencing protocols) off-target effects^83–85^. While the reintroduction of genetically modified cells into human patients will always demand technology that is as perfect as can be humanly obtained, it appears as if CRISPR–Cas9 will be up to that rigorous challenge.

An additional noteworthy variation that came out of attempts to limit the off-target effects of CRISPR–Cas9 was the development of Cas9 variants that only nick genomic DNA (nickase Cas9 or ‘nCas9’)^86^. Thus, if only one of the two active nuclease sites is mutated, the resulting Cas9 still retains the ability to nick one of the two strands. Because nicks are not DSBs (and are probably not frequently converted to DSBs), NHEJ does not act on the broken DNA ends. This in principle (and practice^86^), greatly decreases the possibility of generating mutations at off-target sites. Needless to say, however, nicks, while surprisingly recombinogenic^87^, are not nearly as recombinogenic as DSBs. Thus, the improvement observed for not generating ancillary deleterious mutations is offset by the reduced frequencies of gene editing. However, concerted effort is currently being expended trying to utilize and optimize nick-induced recombination^39,88,89^, and if this can indeed be improved, it certainly is likely to widen its utilization especially in clinical settings^90,91^.

### CRISPR and HMEJ, a novel approach to gene therapy

**Fig. 3:**
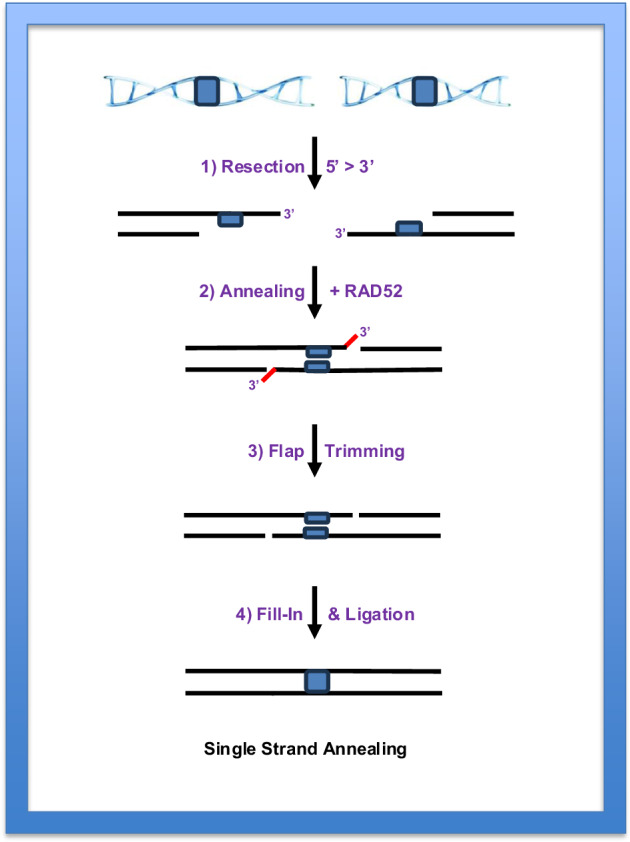
Schematic depicting the SSA pathway. Two regions of homology (blue rectangles) are shown on either side of a DSB. Step 1: a 5′ > 3′ resection occurs at both ends, exposing the homology. Step 2: in a RAD52-dependent fashion, the single-stranded ends are annealed together. Step 3: residual 3′-flaps (red lines) are nucleolytically trimmed away. Step 4: the gaps are filled in with polymerases, and any nicks are ligated to complete the repair process.

Throughout this review, the use of HDR to achieve knock-in gene editing outcomes has been extolled. It is important to remember, however, that besides the major canonical HDR pathway (Fig. 2), which is what is used for all the gene editing hitherto described, there exist additional subpathways of HDR. One of these subpathways, termed single-strand annealing (SSA), was first characterized when measuring recombination between repetitive elements in either mouse^92^ or yeast^93^ cells. SSA also requires extensive resection to uncover regions of homology flanking either side of a single DSB^94,95^ (Fig. 3). These homologies are then annealed in (importantly) an RAD51-independent but radiation sensitive 52 (RAD52)-dependent, fashion (Fig. 3, step 2). This annealing often generates an intermediate with flaps, which need to be trimmed. Finally, the resulting gaps can be filled in by DNA polymerase action and any nicks sealed by DNA ligation (Fig. 3, steps 3 and 4).

### HMEJ and SSA

A decade ago, a series of papers described a method termed ‘precise integration into target chromosome’ (PITCh) in which a DSB was introduced into a chromosome, and a second DSB was introduced onto a donor plasmid carrying short (~20–50 bp) homology to the chromosomal site of interest^96–100^. These studies (performed in multiple species and human cell lines) demonstrated that integration took place at a surprisingly high frequency and with great precision (that is, very few indels), at least at the end harboring the homology. Additional work demonstrated that introducing two DSBs into the vector to liberate a linear dsDNA donor with short homology at both ends, increased the frequency of integration and the precision (now at both ends)^96,101,102^. The CRISPR-mediated linear nature of the donor DNA proved important as the PCR generation of linear dsDNA donors with only 33 bp of homology also worked efficiently^103^. Other investigators repeated these studies, and because they found a requirement for more homology (~300–600 bp) for high frequency integration, they dubbed the process more generically ‘homology-mediated end joining’ (HMEJ)^104,105^. To resolve some of these discrepancies, the Moriarity and Webber laboratories adapted HMEJ and showed that with homology arms as short as 24–48 bp, high frequency (20–100%) gene targeting could be achieved at eight different chromosomal loci in zebrafish^106^. More recently, they also showed that with homology arms of 48 bp that high frequency (generally >30%) integration of donor DNA occurred nearly seamlessly (a remarkable near background from 0.5% to 1.5% indel/mutation frequency at the sites of integration) into the safe-harbor adeno-associated viral integration site 1 locus in activated, primary human T cells^102^. Moreover, they found that HMEJ was well suited for large genetic cargo integration (2.6 kb at >30% and 6.3 kb >20%), which is substantial as advanced genome engineering for immunotherapies requires increasingly more genetic cargo to be effective^102^. In summary, HMEJ works in a variety of organisms and multiple human cell lines and provides a methodology for achieving high frequency, precise genome editing.

**Fig. 4:**
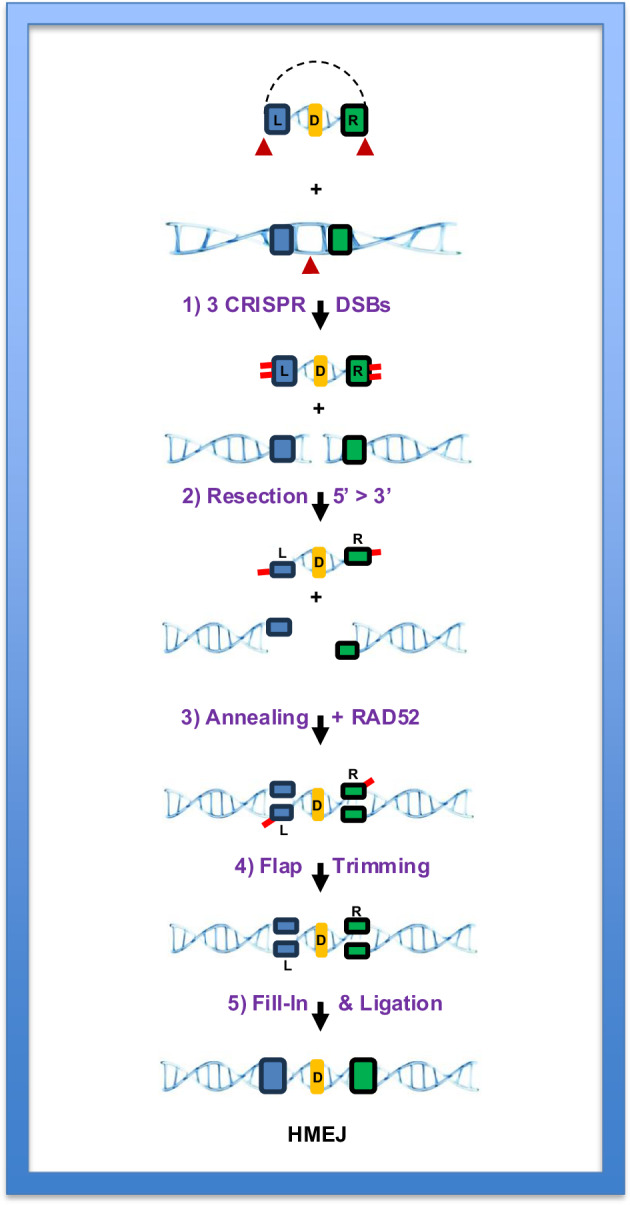
Schematic depicting a hypothetical HMEJ pathway. A donor DNA within a plasmid (dashed lines) contains short (~40 bp) homology arms on the left (blue box, L) and the right (green box, R) of the new genetic information to be incorporated into the chromosome (yellow box, D). Bottom: the chromosomal target region is shown. The sites of CRISPR cleavage are denoted with red arrowheads. Step 1: two CRISPR cuts liberate a linear dsDNA donor, which has short nonhomology at either end (red lines). A third CRISPR-mediated DSB is also introduced on the chromosome at the site where the genetic modification is desired. Step 2: the ends (four in total) of the donor DNA and the chromosome are resected, exposing the regions of homology in ssDNA form. Step 3: in a RAD52-dependent process, the ends are annealed together. Step 4: the flaps are trimmed away. Step 5: the residual gaps are filled in, and the nicks are ligated. This results in the incorporation of the new genetic information (D) onto the chromosome at the desired location in a nonmutagenic, seamless fashion. Note that the similarities between SSA (Fig. 3) and this putative HMEJ mechanism are compelling.

Unsurprisingly, HMEJ has captured the imagination of the gene therapy field. However, how and why HMEJ works so well is unclear. Indeed, HMEJ is mechanistically confusing; it requires much less homology than virtually any canonical HDR-related reaction and yet it generates HDR-like seamless junctions^102^ that are the antithesis of C-NHEJ. We propose that HMEJ is most likely explained as being akin to (but not identical with) SSA. A very similar proposal was also recently put forth by Saito and Adachi^107^. SSA was first described in murine and yeast cells as a process that involved one DSB situated between two repetitive regions (Fig. 3). Theoretically, however, there is no reason why SSA cannot occur when there are multiple DSBs. Importantly too, although the repetitive regions at endogenous loci where SSA has been described are typically hundreds to thousands of base pairs in length, SSA was experimentally shown to occur when the homology was as short as 29 bp^108^. Thus, we hypothesize that the use of CRISPR to produce three DSBs generates a chromosome with a DSB at the site of interest and a linear dsDNA donor with ~40 bp homology arms (Fig. 4, step 1). The relevant four ends (the two on the donor DNA and the two at the chromosomal DSB site) then undergo 5′ > 3′ resection and reveal the regions of homology (Fig. 4, step 2). The single-stranded ends are then annealed in a RAD52-dependent fashion (Fig. 4, step 3) before extraneous flaps are removed and the resulting gaps filled in and ligated (Fig. 4, steps 4 and 5). Hence, on paper, our proposed mechanism for HMEJ bears a striking similarity to the accepted mechanism for SSA, just with additional ends and regions of homology (a comparison of Figs. 3 and 4). An important corollary of this conclusion is that, as a subpathway, HMEJ should not be dependent upon canonical HDR factors such as RAD51 and BRCA1/2, a feature that we and others^107^ have preliminarily confirmed.

The potential clinical importance of HMEJ is related to (1) its error-free nature, (2) its ability to deliver large cargos and (3) its high efficiency. The stated goal of precision medicine is to be able to identify a genetic mutation in an affected individual and then alter that patient’s DNA back to wild type. On paper this seems straightforward. The reality for many disorders (for example, Fanconi anemia, muscular dystrophy and breast cancer), however, is that the gene affected is large and it can be deleteriously impacted by multiple mutations. Thus, from a gene therapy point of view, every patient needs his or her own Food and Drug Administration-certified reagents (for example, Cas9, sgRNAs and donor DNAs) that will have to be meticulously vetted to make sure they are efficacious without undue side effects. This turns out to be complicated, time-consuming and very expensive. Consequently, a therapeutic alternative is to integrate a full-length, wild-type copy of the gene at its corresponding endogenous locus such that the complementary DNA will be expressed using the endogenous promoter at normal levels and with normal timing. This approach (complementation) is amenable to all genes and all mutations that cause a loss-of-function and, as such, can be very broadly applied. The downside of the cDNA complementation strategy is that many genes are large and so are their corresponding cDNAs. This creates a logistical hurdle, as many of the canonical HDR vectors are themselves large and/or can only handle smaller cDNAs making donor DNA delivery virtually unfeasible. Since canonical HDR currently occurs only at low frequency, lowering the amount of donor DNA will only reduce those outcomes to nontherapeutic levels. HMEJ is poised to solve this issue. First, the donor DNAs integrated by HMEJ are imbedded with the same error-free precision (a critical requirement of gene therapy) achieved by canonical HDR. Second, because the HMEJ homology arms are so short, there is much more room for bigger cargo. Indeed, cargos (for example, cDNAs) of over 6 kb can be effectively integrated using HMEJ^102^. Although there will probably be an upper limit to the size of HMEJ donors, 6 kb is already large enough to encode most human genes. Third, the frequency of HMEJ is surprisingly high (certainly well above therapeutic requirements) and often surpasses that of canonical HDR^102,107^. Why this is the case and whether this will turn out to be true for all human tissues is not clear, but given the preliminary success with HMEJ, many more studies will be carried out in the near future.

## Summary

In the four decades that have elapsed since the first report of a gene targeting study in human somatic cells^13^, many endogenous genes have been disrupted or modified. Progress in this area, as is often true in many areas of science, was painfully slow such that after the first two decades only a handful of genes had been modified^41,109^. In the past decade, however, the field has seen spectacular technological progress, with the development of CRISPR–Cas9 being the slam-dunk exclamation point. Thus, as the promise of the CRISPR–Cas9 systems come to fruition, researchers working on human cells in culture will have a powerful and facile weapon at their scientific disposal.

With this said, we would be remiss if we did not mention some of the obstacles still standing in the way of making CRISPR-mediated gene therapy as common as a flu shot. First, we have already described the issue of off-target nicks and breaks mediated by Cas9. While many of these can be avoided through the use of evolved (and greatly improved) Cas9 proteins and optimized sgRNAs, it is probably going to be impossible to completely obviate some ancillary damage. Trying to maximally identify, characterize and project the potential deleterious outcomes of such off-target effects is still a very active area of research. Second, we have also briefly touched upon the subject of how delivery of the reagents themselves (for example, in the form of dsDNA) can cause adverse immunological reactions. Layered on top of this though is the technical limitations of reagent delivery to a specific organ. CRISPR-mediated gene therapy for the eye has already achieved some rather spectacular success^110,111^. In this particular instance, however, the regents can be injected into the eye’s retina, achieving a high concentration of localized delivery. Similarly, blood-related disorders can be addressed by removing hematopoietic stem cells from a patient’s bone marrow, manipulating them in tissue culture and then reintroducing them back into the patient^112,113^. This can be achieved with high efficiency and specificity because of the robustness of the bone marrow transplant procedure. Most organs, however, such as the heart, lungs, brain and so on do not easily lend themselves to gene therapeutic approaches. Thus, a huge amount of effort is being directed into optimized delivery platforms that utilize improved virological, biomedically engineered liposomes/biomaterials or nanotechnological-based approaches to achieve targeted, high efficiency delivery of gene editing reagents^114,115^. Third, a very practical concern is cost. In every instance where gene therapy reagents or ex vivo genetically modified cells will be introduced back into patients virtually every step and reagent in those protocols must be quality controlled at good manufacturing practice standards. Moreover, since many diseases are caused not by a single, reoccurring mutation in the population but by a myriad of different mutations (there are, for example, at least 1600 and 1800 known pathogenic mutations in the BRCA1 and BRCA2 genes, respectively^116^) this means that a novel set of extremely expensive reagents must be developed for each and every patient. A fourth obstacle is ethics. It seems eminently clear that treating someone suffering from a debilitating disease that is likely to cause them significant suffering and loss of life is an ethically justifiable endeavor. Alas, as a myriad of ethicists have pointed out, this slope is slippery^117^. Thus, if CRISPR-mediated gene therapy is ever truly optimized and the technology is literally there for use, it is only a matter of time before someone will avail themselves of it in an attempt to make a ‘designer genome’. By today’s standards, this is clearly an unjustifiable endeavor^118^.

Finally, it should be emphasized that this review has cherry picked topics and applications for discussion. We have not, for example, mentioned CRISPR–Cas9-mediated base editing^90^ nor ‘prime’ editing^91^, subjects for which an enormous literature and clinical interest exists. Besides these, there are many other uses, permutations and applications for CRISPR–Cas9, and the reader is enthusiastically encouraged to explore other excellent reviews that may better address topics that are nearer and dearer to their scientific hearts.

## References

[1] Southern, E. Southern blotting. *Nat. Protoc.* **1**, 518–525 (2006). 17406277 10.1038/nprot.2006.73

[2] Fields, S. & Song, O. A novel genetic system to detect protein-protein interactions. *Nature* **340**, 245–246 (1989). 2547163 10.1038/340245a0

[3] Kohler, G. & Milstein, C. Continuous cultures of fused cells secreting antibody of predefined specificity. *Nature* **256**, 495–497 (1975). 1172191 10.1038/256495a0

[4] Saiki, R. K. et al. Enzymatic amplification of beta-globin genomic sequences and restriction site analysis for diagnosis of sickle cell anemia. *Science* **230**, 1350–1354 (1985). 2999980 10.1126/science.2999980

[5] Cong, L. et al. Multiplex genome engineering using CRISPR/Cas systems. *Science* **339**, 819–823 (2013). 23287718 10.1126/science.1231143 PMC3795411

[6] Jinek, M. et al. RNA-programmed genome editing in human cells. *eLife* **2**, e00471 (2013). 23386978 10.7554/eLife.00471 PMC3557905

[7] Mali, P. et al. RNA-guided human genome engineering via Cas9. *Science* **339**, 823–826 (2013). 23287722 10.1126/science.1232033 PMC3712628

[8] Liang, P. et al. CRISPR/Cas9-mediated gene editing in human tripronuclear zygotes. *Protein Cell* **6**, 363–372 (2015). 25894090 10.1007/s13238-015-0153-5 PMC4417674

[9] Baltimore, D. et al. Biotechnology. A prudent path forward for genomic engineering and germline gene modification. *Science* **348**, 36–38 (2015). 25791083 10.1126/science.aab1028 PMC4394183

[10] Travis, J. Genetic engineering. Germline editing dominates DNA summit. *Science* **350**, 1299–1300 (2015). 26659031 10.1126/science.350.6266.1299

[11] Lopes, R. & Prasad, M. K. Beyond the promise: evaluating and mitigating off-target effects in CRISPR gene editing for safer therapeutics. *Front. Bioeng. Biotechnol.* **11**, 1339189 (2023). 38390600 10.3389/fbioe.2023.1339189 PMC10883050

[12] Shumega, A. R. et al. CRISPR/Cas9 as a mutagenic factor. *Int. J. Mol. Sci.* **25**, 823 (2024). 38255897 10.3390/ijms25020823 PMC10815272

[13] Smithies, O. et al. Insertion of DNA sequences into the human chromosomal beta-globin locus by homologous recombination. *Nature* **317**, 230–234 (1985). 2995814 10.1038/317230a0

[14] Jasin, M. et al. Gene targeting at the human CD4 locus by epitope addition. *Genes Dev.* **4**, 157–166 (1990). 1692556 10.1101/gad.4.2.157

[15] Khan, I. F., Hirata, R. K. & Russell, D. W. AAV-mediated gene targeting methods for human cells. *Nat. Protoc.* **6**, 482–501 (2011). 21455185 10.1038/nprot.2011.301 PMC3739714

[16] Xiao, P. J., Lentz, T. B. & Samulski, R. J. Recombinant adeno-associated virus: clinical application and development as a gene-therapy vector. *Ther. Deliv.* **3**, 835–856 (2012). 22900466 10.4155/tde.12.63

[17] Rouet, P., Smih, F. & Jasin, M. Introduction of double-strand breaks into the genome of mouse cells by expression of a rare-cutting endonuclease. *Mol. Cell Biol.* **14**, 8096–8106 (1994). 7969147 10.1128/mcb.14.12.8096-8106.1994 PMC359348

[18] Smih, F. et al. Double-strand breaks at the target locus stimulate gene targeting in embryonic stem cells. *Nucleic Acids Res.* **23**, 5012–5019 (1995). 8559659 10.1093/nar/23.24.5012 PMC307507

[19] Carroll, D. Genome engineering with zinc-finger nucleases. *Genetics* **188**, 773–782 (2011). 21828278 10.1534/genetics.111.131433 PMC3176093

[20] Bogdanove, A. J. & Voytas, D. F. TAL effectors: customizable proteins for DNA targeting. *Science* **333**, 1843–1846 (2011). 21960622 10.1126/science.1204094

[21] Wright, A. V., Nunez, J. K. & Doudna, J. A. Biology and applications of CRISPR systems: harnessing nature’s toolbox for genome engineering. *Cell* **164**, 29–44 (2016). 26771484 10.1016/j.cell.2015.12.035

[22] Chang, H. H. Y. et al. Non-homologous DNA end joining and alternative pathways to double-strand break repair. *Nat. Rev. Mol. Cell Biol.* **18**, 495–506 (2017). 28512351 10.1038/nrm.2017.48 PMC7062608

[23] Krenning, L., van den Berg, J. & Medema, R. H. Life or death after a break: what determines the choice? *Mol. Cell* **76**, 346–358 (2019). 31561953 10.1016/j.molcel.2019.08.023

[24] Roth, D. B. & Wilson, J. H. Relative rates of homologous and nonhomologous recombination in transfected DNA. *Proc. Natl Acad. Sci. USA* **82**, 3355–3359 (1985). 2987922 10.1073/pnas.82.10.3355 PMC397774

[25] Thomas, K. R. & Capecchi, M. R. Site-directed mutagenesis by gene targeting in mouse embryo-derived stem cells. *Cell* **51**, 503–512 (1987). 2822260 10.1016/0092-8674(87)90646-5

[26] Lee, S. E. et al. Evidence for DNA-PK-dependent and -independent DNA double-strand break repair pathways in mammalian cells as a function of the cell cycle. *Mol. Cell Biol.* **17**, 1425–1433 (1997). 9032269 10.1128/mcb.17.3.1425 PMC231867

[27] Takata, M. et al. Homologous recombination and non-homologous end-joining pathways of DNA double-strand break repair have overlapping roles in the maintenance of chromosomal integrity in vertebrate cells. *EMBO J.* **17**, 5497–5508 (1998). 9736627 10.1093/emboj/17.18.5497 PMC1170875

[28] Hui, Z. et al. Development and therapeutic potential of DNA-dependent protein kinase inhibitors. *Bioorg. Chem.* **150**, 107608 (2024). 38981210 10.1016/j.bioorg.2024.107608

[29] Goff, N. J. et al. DNA-PK: a synopsis beyond synapsis. *DNA Repair* **141**, 103716 (2024). 38996771 10.1016/j.dnarep.2024.103716 PMC11369974

[30] Iliakis, G. Backup pathways of NHEJ in cells of higher eukaryotes: cell cycle dependence. *Radiother. Oncol.* **92**, 310–315 (2009). 19604590 10.1016/j.radonc.2009.06.024

[31] McVey, M. & Lee, S. E. MMEJ repair of double-strand breaks (director’s cut): deleted sequences and alternative endings. *Trends Genet.* **24**, 529–538 (2008). 18809224 10.1016/j.tig.2008.08.007 PMC5303623

[32] Sfeir, A. & Symington, L. S. Microhomology-mediated end joining: a back-up survival mechanism or dedicated pathway? *Trends Biochem. Sci.* **40**, 701–714 (2015). 26439531 10.1016/j.tibs.2015.08.006 PMC4638128

[33] Bhargava, R. et al. C-NHEJ without indels is robust and requires synergistic function of distinct XLF domains. *Nat. Commun.* **9**, 2484 (2018). 29950655 10.1038/s41467-018-04867-5 PMC6021437

[34] Gelot, C. et al. Poltheta is phosphorylated by PLK1 to repair double-strand breaks in mitosis. *Nature* **621**, 415–422 (2023). 37674080 10.1038/s41586-023-06506-6 PMC10499603

[35] Ramsden, D. A., Carvajal-Garcia, J. & Gupta, G. P. Mechanism, cellular functions and cancer roles of polymerase-theta-mediated DNA end joining. *Nat. Rev. Mol. Cell Biol.* **23**, 125–140 (2022). 34522048 10.1038/s41580-021-00405-2 PMC13537726

[36] Jasin, M. & Rothstein, R. Repair of strand breaks by homologous recombination. *Cold Spring Harb. Perspect. Biol.* **5**, a012740 (2013). 24097900 10.1101/cshperspect.a012740 PMC3809576

[37] Bonilla, B. et al. RAD51 gene family structure and function. *Annu. Rev. Genet.* **54**, 25–46 (2020). 32663049 10.1146/annurev-genet-021920-092410 PMC7703940

[38] Kan, Y. et al. The mechanism of gene targeting in human somatic cells. *PLoS Genet.* **10**, e1004251 (2014). 24699519 10.1371/journal.pgen.1004251 PMC3974634

[39] Kan, Y. et al. Mechanisms of precise genome editing using oligonucleotide donors. *Genome Res.* **27**, 1099–1111 (2017). 28356322 10.1101/gr.214775.116 PMC5495063

[40] Orthwein, A. et al. A mechanism for the suppression of homologous recombination in G1 cells. *Nature* **528**, 422–426 (2015). 26649820 10.1038/nature16142 PMC4880051

[41] Hendrickson, E. A. in *Source Book of Models for Biomedical Research* (ed. Conn, P. M.) 509–525 (Humana Press, 2008).

[42] Cong, L. & Zhang, F. Genome engineering using CRISPR-Cas9 system. *Methods Mol. Biol.* **1239**, 197–217 (2015). 25408407 10.1007/978-1-4939-1862-1_10

[43] Myers, C. N. et al. Mutant versions of the *S. cerevisiae* transcription elongation factor Spt16 define regions of Spt16 that functionally interact with histone H3. *PLoS ONE* **6**, e20847 (2011). 21673966 10.1371/journal.pone.0020847 PMC3108975

[44] Szappanos, B. et al. An integrated approach to characterize genetic interaction networks in yeast metabolism. *Nat. Genet.* **43**, 656–662 (2011). 21623372 10.1038/ng.846 PMC3125439

[45] Piotrowski, J. S. et al. Chemical genomic profiling via barcode sequencing to predict compound mode of action. *Methods Mol. Biol.* **1263**, 299–318 (2015). 25618354 10.1007/978-1-4939-2269-7_23 PMC4497522

[46] Wang, T. et al. Genetic screens in human cells using the CRISPR–Cas9 system. *Science* **343**, 80–84 (2014). 24336569 10.1126/science.1246981 PMC3972032

[47] Shalem, O. et al. Genome-scale CRISPR–Cas9 knockout screening in human cells. *Science* **343**, 84–87 (2014). 24336571 10.1126/science.1247005 PMC4089965

[48] Parnas, O. et al. A genome-wide CRISPR screen in primary immune cells to dissect regulatory networks. *Cell* **162**, 675–686 (2015). 26189680 10.1016/j.cell.2015.06.059 PMC4522370

[49] Boone, C. & Andrews, B. J. Human Genome. The indispensable genome. *Science* **350**, 1028–1029 (2015). 26612934 10.1126/science.aad7925

[50] Wang, T. et al. Identification and characterization of essential genes in the human genome. *Science* **350**, 1096–1101 (2015). 26472758 10.1126/science.aac7041 PMC4662922

[51] Wang, H. et al. One-step generation of mice carrying mutations in multiple genes by CRISPR/Cas-mediated genome engineering. *Cell* **153**, 910–918 (2013). 23643243 10.1016/j.cell.2013.04.025 PMC3969854

[52] Yang, L. et al. Genome-wide inactivation of porcine endogenous retroviruses (PERVs). *Science* **350**, 1101–1104 (2015). 26456528 10.1126/science.aad1191

[53] Hurley, P. J., Wilsker, D. & Bunz, F. Human cancer cells require ATR for cell cycle progression following exposure to ionizing radiation. *Oncogene* **26**, 2535–2542 (2007). 17043640 10.1038/sj.onc.1210049

[54] Cui, X. et al. Targeted integration in rat and mouse embryos with zinc-finger nucleases. *Nat. Biotechnol.* **29**, 64–67 (2011). 21151125 10.1038/nbt.1731

[55] Miller, J. C. et al. A TALE nuclease architecture for efficient genome editing. *Nat. Biotechnol.* **29**, 143–148 (2011). 21179091 10.1038/nbt.1755

[56] Yang, H. et al. One-step generation of mice carrying reporter and conditional alleles by CRISPR/Cas-mediated genome engineering. *Cell* **154**, 1370–1379 (2013). 23992847 10.1016/j.cell.2013.08.022 PMC3961003

[57] Chu, V. T. et al. Increasing the efficiency of homology-directed repair for CRISPR–Cas9-induced precise gene editing in mammalian cells. *Nat. Biotechnol.* **33**, 543–548 (2015). 25803306 10.1038/nbt.3198

[58] Maruyama, T. et al. Increasing the efficiency of precise genome editing with CRISPR–Cas9 by inhibition of nonhomologous end joining. *Nat. Biotechnol.* **33**, 538–542 (2015). 25798939 10.1038/nbt.3190 PMC4618510

[59] Song, J. et al. RS-1 enhances CRISPR/Cas9- and TALEN-mediated knock-in efficiency. *Nat. Commun.* **7**, 10548 (2016). 26817820 10.1038/ncomms10548 PMC4738357

[60] Wilde, J. J. et al. Efficient embryonic homozygous gene conversion via RAD51-enhanced interhomolog repair. *Cell* **184**, 3267–3280.e18 (2021). 34043941 10.1016/j.cell.2021.04.035 PMC8240950

[61] Leal, A. F. et al. Current strategies for increasing knock-in efficiency in CRISPR/Cas9-based approaches. *Int. J. Mol. Sci.* **25**, 2456 (2024). 38473704 10.3390/ijms25052456 PMC10931195

[62] Shakirova, A. et al. In search of an ideal template for therapeutic genome editing: a review of current developments for structure optimization. *Front. Genome Ed.* **5**, 1068637 (2023). 36911237 10.3389/fgeed.2023.1068637 PMC9992834

[63] Brizzard, B. Epitope tagging. *Biotechniques* **44**, 693–695 (2008). 18474046 10.2144/000112841

[64] Chung, H. K. et al. Tunable and reversible drug control of protein production via a self-excising degron. *Nat. Chem. Biol.* **11**, 713–720 (2015). 26214256 10.1038/nchembio.1869 PMC4543534

[65] Vandemoortele, G., Gevaert, K. & Eyckerman, S. Proteomics in the genome engineering era. *Proteomics* **16**, 177–187 (2016). 26510734 10.1002/pmic.201500262

[66] Nabet, B. et al. The dTAG system for immediate and target-specific protein degradation. *Nat. Chem. Biol.* **14**, 431–441 (2018). 29581585 10.1038/s41589-018-0021-8 PMC6295913

[67] Lackner, D. H. et al. A generic strategy for CRISPR–Cas9-mediated gene tagging. *Nat. Commun.* **6**, 10237 (2015). 26674669 10.1038/ncomms10237 PMC4703899

[68] Vandemoortele, G., Eyckerman, S. & Gevaert, K. Pick a tag and explore the functions of your pet protein. *Trends Biotechnol.* **37**, 1078–1090 (2019). 31036349 10.1016/j.tibtech.2019.03.016

[69] Sternberg, S. H. et al. Conformational control of DNA target cleavage by CRISPR–Cas9. *Nature* **527**, 110–113 (2015). 26524520 10.1038/nature15544 PMC4859810

[70] Larson, M. H. et al. CRISPR interference (CRISPRi) for sequence-specific control of gene expression. *Nat. Protoc.* **8**, 2180–2196 (2013). 24136345 10.1038/nprot.2013.132 PMC3922765

[71] Qi, L. S. et al. Repurposing CRISPR as an RNA-guided platform for sequence-specific control of gene expression. *Cell* **152**, 1173–1183 (2013). 23452860 10.1016/j.cell.2013.02.022 PMC3664290

[72] Clark, T. et al. CRISPR activation screens: navigating technologies and applications. *Trends Biotechnol.* **42**, 1017–1034 (2024). 38493051 10.1016/j.tibtech.2024.02.007

[73] Ilyas, M. et al. Advances in CRISPR–Cas systems for epigenetics. *Prog. Mol. Biol. Transl. Sci.* **208**, 185–209 (2024). 39266182 10.1016/bs.pmbts.2024.07.003

[74] Dominguez, A. A., Lim, W. A. & Qi, L. S. Beyond editing: repurposing CRISPR-Cas9 for precision genome regulation and interrogation. *Nat. Rev. Mol. Cell Biol.* **17**, 5–15 (2016). 26670017 10.1038/nrm.2015.2 PMC4922510

[75] Zou, R. S. et al. Cas9 deactivation with photocleavable guide RNAs. *Mol. Cell* **81**, 1553–1565.e8 (2021). 33662274 10.1016/j.molcel.2021.02.007 PMC8026597

[76] Fu, Y. et al. High-frequency off-target mutagenesis induced by CRISPR-Cas nucleases in human cells. *Nat. Biotechnol.* **31**, 822–826 (2013). 23792628 10.1038/nbt.2623 PMC3773023

[77] Hsu, P. D. et al. DNA targeting specificity of RNA-guided Cas9 nucleases. *Nat. Biotechnol.* **31**, 827–832 (2013). 23873081 10.1038/nbt.2647 PMC3969858

[78] Pattanayak, V. et al. High-throughput profiling of off-target DNA cleavage reveals RNA-programmed Cas9 nuclease specificity. *Nat. Biotechnol.* **31**, 839–843 (2013). 23934178 10.1038/nbt.2673 PMC3782611

[79] Doench, J. G. et al. Rational design of highly active sgRNAs for CRISPR–Cas9-mediated gene inactivation. *Nat. Biotechnol.* **32**, 1262–1267 (2014). 25184501 10.1038/nbt.3026 PMC4262738

[80] Knight, S. C. et al. Dynamics of CRISPR–Cas9 genome interrogation in living cells. *Science* **350**, 823–826 (2015). 26564855 10.1126/science.aac6572

[81] Sternberg, S. H. et al. DNA interrogation by the CRISPR RNA-guided endonuclease Cas9. *Nature* **507**, 62–67 (2014). 24476820 10.1038/nature13011 PMC4106473

[82] Tsai, S. Q. et al. GUIDE-seq enables genome-wide profiling of off-target cleavage by CRISPR–Cas nucleases. *Nat. Biotechnol.* **33**, 187–197 (2015). 25513782 10.1038/nbt.3117 PMC4320685

[83] Kleinstiver, B. P. et al. High-fidelity CRISPR–Cas9 nucleases with no detectable genome-wide off-target effects. *Nature* **529**, 490–495 (2016). 26735016 10.1038/nature16526 PMC4851738

[84] Slaymaker, I. M. et al. Rationally engineered Cas9 nucleases with improved specificity. *Science* **351**, 84–88 (2016). 26628643 10.1126/science.aad5227 PMC4714946

[85] Urnov, F. Genome editing: the domestication of Cas9. *Nature* **529**, 468–469 (2016). 26819037 10.1038/529468a

[86] Ran, F. A. et al. Double nicking by RNA-guided CRISPR Cas9 for enhanced genome editing specificity. *Cell* **154**, 1380–1389 (2013). 23992846 10.1016/j.cell.2013.08.021 PMC3856256

[87] Lee, G. S. et al. RAG proteins shepherd double-strand breaks to a specific pathway, suppressing error-prone repair, but RAG nicking initiates homologous recombination. *Cell* **117**, 171–184 (2004). 15084256 10.1016/s0092-8674(04)00301-0

[88] Richardson, C. D. et al. Enhancing homology-directed genome editing by catalytically active and inactive CRISPR–Cas9 using asymmetric donor DNA. *Nat Biotechnol.* **34**, 339–344 (2016). 10.1038/nbt.3481 26789497

[89] Nan, A. X. et al. Ligase-mediated programmable genomic integration (L-PGI): an efficient site-specific gene editing system that overcomes the limitations of reverse transcriptase-based editing systems. Preprint at *bioRxiv* 2024. 10.1101/2024.09.27.615478.

[90] Rees, H. A. & Liu, D. R. Base editing: precision chemistry on the genome and transcriptome of living cells. *Nat. Rev. Genet* **19**, 770–788 (2018). 30323312 10.1038/s41576-018-0059-1 PMC6535181

[91] Chen, P. J. & Liu, D. R. Prime editing for precise and highly versatile genome manipulation. *Nat. Rev. Genet* **24**, 161–177 (2023). 36344749 10.1038/s41576-022-00541-1 PMC10989687

[92] Lin, F. L., Sperle, K. & Sternberg, N. Model for homologous recombination during transfer of DNA into mouse L cells: role for DNA ends in the recombination process. *Mol. Cell Biol.* **4**, 1020–1034 (1984). 6330525 10.1128/mcb.4.6.1020-1034.1984 PMC368869

[93] Ivanov, E. L. et al. Genetic requirements for the single-strand annealing pathway of double-strand break repair in Saccharomyces cerevisiae. *Genetics* **142**, 693–704 (1996). 8849880 10.1093/genetics/142.3.693 PMC1207011

[94] Bhargava, R., Onyango, D. O. & Stark, J. M. Regulation of single-strand annealing and its role in genome maintenance. *Trends Genet.* **32**, 566–575 (2016). 27450436 10.1016/j.tig.2016.06.007 PMC4992407

[95] Blasiak, J. Single-strand annealing in cancer. *Int. J. Mol. Sci.* **22**, 2167 (2021). 33671579 10.3390/ijms22042167 PMC7926775

[96] Nakade, S. et al. Microhomology-mediated end-joining-dependent integration of donor DNA in cells and animals using TALENs and CRISPR/Cas9. *Nat. Commun.* **5**, 5560 (2014). 25410609 10.1038/ncomms6560 PMC4263139

[97] Hisano, Y. et al. Precise in-frame integration of exogenous DNA mediated by CRISPR/Cas9 system in zebrafish. *Sci. Rep.* **5**, 8841 (2015). 25740433 10.1038/srep08841 PMC4350073

[98] Sakuma, T. et al. MMEJ-assisted gene knock-in using TALENs and CRISPR–Cas9 with the PITCh systems. *Nat. Protoc.* **11**, 118–133 (2016). 26678082 10.1038/nprot.2015.140

[99] Sakuma, T. et al. Homologous recombination-independent large gene cassette knock-in in CHO cells using TALEN and MMEJ-directed donor plasmids. *Int. J. Mol. Sci.* **16**, 23849–23866 (2015). 26473830 10.3390/ijms161023849 PMC4632728

[100] Aida, T. et al. Gene cassette knock-in in mammalian cells and zygotes by enhanced MMEJ. *BMC Genomics* **17**, 979 (2016). 27894274 10.1186/s12864-016-3331-9 PMC5126809

[101] Kanca, O. et al. An efficient CRISPR-based strategy to insert small and large fragments of DNA using short homology arms. *eLife* **8**, e51539 (2019). 10.7554/eLife.51539 PMC6855806 31674908

[102] Webber, B. R. et al. Cas9-induced targeted integration of large DNA payloads in primary human T cells via homology-mediated end-joining DNA repair. *Nat. Biomed. Eng.* **8**, 1553–1570 (2023). 10.1038/s41551-023-01157-4 PMC11169092 38092857

[103] Paix, A. et al. Precision genome editing using synthesis-dependent repair of Cas9-induced DNA breaks. *Proc. Natl Acad. Sci. USA* **114**, E10745–E10754 (2017). 29183983 10.1073/pnas.1711979114 PMC5740635

[104] Yao, X. et al. Homology-mediated end joining-based targeted integration using CRISPR/Cas9. *Cell Res.* **27**, 801–814 (2017). 28524166 10.1038/cr.2017.76 PMC5518881

[105] Zhang, J. P. et al. Efficient precise knockin with a double cut HDR donor after CRISPR/Cas9-mediated double-stranded DNA cleavage. *Genome Biol.* **18**, e35 (2017). 10.1186/s13059-017-1164-8 PMC5319046 28219395

[106] Wierson, W. A. et al. Efficient targeted integration directed by short homology in zebrafish and mammalian cells. *eLife* **9**, e53968 (2020). 10.7554/eLife.53968 PMC7228771 32412410

[107] Saito, S. & Adachi, N. Characterization and regulation of cell cycle-independent noncanonical gene targeting. *Nat. Commun.* **15**, 5044 (2024). 38890315 10.1038/s41467-024-49385-9 PMC11189520

[108] Sugawara, N., Ira, G. & Haber, J. E. DNA length dependence of the single-strand annealing pathway and the role of *Saccharomyces cerevisiae* RAD59 in double-strand break repair. *Mol. Cell Biol.* **20**, 5300–5309 (2000). 10866686 10.1128/mcb.20.14.5300-5309.2000 PMC85979

[109] Sedivy, J. M. et al. Gene targeting in human cells without isogenic DNA. *Science* **283**, 9a (1999).

[110] Ahmad, I. CRISPR/Cas9-a promising therapeutic tool to cure blindness: current scenario and future prospects. *Int. J. Mol. Sci.* **23**, e11482 (2022). 10.3390/ijms231911482 PMC9569777 36232782

[111] Pierce, E. A. et al. Gene editing for CEP290-associated retinal degeneration. *N. Engl. J. Med* **390**, 1972–1984 (2024). 38709228 10.1056/NEJMoa2309915 PMC11389875

[112] George, C. A. et al. Genome editing therapy for the blood: ex vivo success and in vivo prospects. *CRISPR J.* **7**, 231–248 (2024). 39324895 10.1089/crispr.2024.0036

[113] Mahadevia, H. et al. A review on disease modifying pharmacologic therapies for sickle cell disease. *Ann. Hematol.* **104**, 881–893 (2025). 39828781 10.1007/s00277-025-06216-1 PMC11971234

[114] Li, J., Wang, J. & Chen, Z. Emerging role of exosomes in cancer therapy: progress and challenges. *Mol. Cancer* **24**, e13 (2025). 10.1186/s12943-024-02215-4 PMC11727182 39806451

[115] Zhu, C., Mu, J. & Liang, L. Nanocarriers for intracellular delivery of proteins in biomedical applications: strategies and recent advances. *J. Nanobiotechnol.* **22**, 688 (2024). 10.1186/s12951-024-02969-5 PMC11552240 39523313

[116] Lee, A., Moon, B. I. & Kim, T. H. BRCA1/BRCA2 pathogenic variant breast cancer: treatment and prevention strategies. *Ann. Lab. Med.* **40**, 114–121 (2020). 31650727 10.3343/alm.2020.40.2.114 PMC6822003

[117] Bateman-House, A. Somatic gene therapy: ethics and access. *Annu. Rev. Genomics Hum. Genet.* **25**, 421–438 (2024). 39190912 10.1146/annurev-genom-021623-104458

[118] Normile, D. Shock greets claim of CRISPR-edited babies. *Science* **362**, 978–979 (2018). 30498103 10.1126/science.362.6418.978

[119] Shy, B. R. et al. High-yield genome engineering in primary cells using a hybrid ssDNA repair template and small-molecule cocktails. *Nat. Biotechnol.* **41**, 521–531 (2023). 10.1038/s41587-022-01418-8 PMC10065198 36008610

